# Paediatric Acute Kidney Injury in a Tertiary Hospital in Nigeria: Prevalence, Causes and Mortality Rate

**DOI:** 10.1371/journal.pone.0051229

**Published:** 2012-12-10

**Authors:** Christopher Imokhuede Esezobor, Taiwo Augustina Ladapo, Babayemi Osinaike, Foluso Ebun Afolabi Lesi

**Affiliations:** 1 Department of Paediatrics, College of Medicine, University of Lagos, Idi-Araba, Lagos State, Nigeria; 2 Department of Paediatrics, Lagos University Teaching Hospital, Idi-Araba, Lagos State, Nigeria; University of São Paulo School of Medicine, Brazil

## Abstract

**Background:**

The modest decline in child mortality in Africa raises the question whether the pattern of diseases associated with acute kidney injury (AKI) in children in Nigeria has changed.

**Methods:**

A database of children, aged between one month and 16 years, with AKI (using modified pediatric RIFLE criteria) was reviewed. The cause of AKI was defined as the major underlying disease. The clinical and laboratory features of children with AKI who survived were compared to those who died.

**Results:**

Of the 4 015 children admitted into Lagos University Teaching Hospital between July 2010 and July 2012, 70 episodes of AKI were recorded equalling 17.4 cases per 1000 children. The median age of the children with AKI was 4.8 (range 0.1–14.4) years and 68.6% were males. Acute kidney injury was present in 58 (82.9%) children at admission with 70% in ‘failure’ category. Primary kidney disease (38.6%), sepsis (25.7%) and malaria (11.4%) were the commonest causes. The primary kidney diseases were acute glomerulonephritis (11) and nephrotic syndrome (8). Nineteen (28.4%) children with AKI died. Need for dialysis [odds ratio: 10.04 (2.94–34.33)], white cell >15 000/mm^3^ [odds ratio: 5.72 (1.65–19.89)] and platelet <100 000/mm^3^ [odds ratio: 9.56 (2.63–34.77)] were associated with death.

**Conclusion:**

Acute kidney injury is common in children admitted to hospitals. The common causes remain primary kidney diseases, sepsis and malaria but the contribution of sepsis is rising while malaria and gastroenteritis are declining. Acute kidney injury-related mortality remains high.

## Introduction

Acute kidney injury (AKI), formerly known as acute renal failure is common in children admitted to hospitals. [1], [2] Studies from both developing and developed regions of the world have demonstrated high incidence of AKI in children. [1], [3] Significantly, AKI is associated with high morbidity and mortality, more so in regions where there is dearth of resources including renal replacement therapy. [1], [4], [5] In contrast to developed countries where AKI is more common in older children admitted to intensive care units with multiple co-morbidities and multi-organ failure, previous studies of AKI in children in developing countries, documented single disease entities such as diarrhoeal diseases, malaria, haemolytic uremic syndrome and acute glomerulonephritis as the major causes of AKI. [3], [6], [7], [8] Acute kidney injury from single disease entities portends better prognosis compared to AKI following multi-organ failure, however, in developing countries AKI from these causes still carries significant mortality. [3], [4], [5], [6], [7].

Access to dialysis remains poor in developing countries and has contributed to the high morbidity and mortality in studies from these regions. [6], [9] Similarly, studies from Nigeria were consistent in identifying single-disease entities as common causes of AKI, poor access to dialysis and high mortality rates from AKI. [4], [5].

There are no recent studies documenting the prevalence of AKI in children in sub-Saharan Africa despite sustained modest decline in childhood mortality in the past couple of decades; has the spectrum of diseases causing AKI in the region changed? [10] A common feature of publications on AKI in children in the region was the lack of a uniform definition of AKI which makes review and comparison difficult [4], [5], [6]; hence a need to describe AKI using the recent modified pediatric RIFLE criteria (pRIFLE). [11] A third objective of the study was to determine the mortality among children with AKI.

## Methods

### Ethics Statement

Ethical clearance was obtained from the Health Research and Ethics Committee of Lagos University Teaching Hospital before commencement of the study. Informed consent was not obtained because the study was a review (retrospective study) of anonymized data not traceable to the subjects.

### Study Location

The study was carried out in the Lagos University Teaching Hospital (LUTH), located in Lagos State, south west Nigeria. The hospital is a 760-bed tertiary hospital and one of two government-funded tertiary hospitals providing renal care to children in Lagos and neighbouring states. It has about 20 and 65 beds in the emergency room and general wards respectively for children older than one month. The majority of critically ill children are managed in the general wards rather than in the intensive care unit (ICU) of the hospital; mostly due to lack of money and unavailability of ventilator support for young children.

### Data Collection

From July 2010 the paediatric nephrology unit of the hospital started a database of all children managed for AKI. The present study is a review of the database for all children aged one month to 16 years managed for AKI between July 2010 and July 2012; a period of 25 months. During this period about four weeks was lost to industrial action by various cadres of the hospital’s workforce. Acute kidney injury was defined using the pRIFLE criteria [11], i.e. urine output <0.5 ml/kg/hour for greater than eight hours and or an estimated creatinine clearance (eCCl) (using Schwartz’s formula) [12] decrease of at least 25%; If previous eCCl was unavailable a baseline eCCl of 100 ml/min/1.73 m^2^ was assumed. [11] Also, the severity of the AKI was adjudged using the pRIFLE criteria, as ‘R’ for risk, ‘I’ for injury and ‘F’ for failure.

The unit receives referrals from the wards and emergency centre when a child presents or develops clinical features and or laboratory parameters suggestive of a kidney disease. These features includes facial or generalized body swelling; kidney or bladder mass; reduced urine output; passage of dark or blood stained urine; history of prior kidney disease; hypertension; elevated or rising serum creatinine; metabolic acidosis, hyperkalemia and hyponatraemia.

Standard management of managing AKI in the unit includes obtaining a relevant clinical history and performing a focused clinical examination. All children get a complete blood count with blood film and serum electrolytes, urea and creatinine at admission into the hospital. In children with AKI or suspected AKI, serum electrolytes, urea and creatinine are repeated as needed. Being a malaria endemic-region blood film for malaria parasite is performed for children suspected of having malaria. Similarly, serology for hepatitis B, C and HIV is also done for children with AKI. Urinalysis, including microscopy is routinely performed in children making urine. An ultrasound examination of the kidneys, ureters and bladder is done for children suspected of having obstructive uropathy or acute glomerulonephritis.

Indications for renal replacement therapy in the unit include the presence of one or more of the following clinical or laboratory features: pulmonary oedema in the presence of oliguria or anuria, difficult to control severe hypertension, features of uraemia such as alteration in level of consciousness, seizures or bleeding diathesis, intractable metabolic acidosis especially with serum bicarbonate <12 mmol/l and severe hyperkalemia (serum potassium >7.0 mmol/l). Peritoneal dialysis is offered to children weighing less than 25 kg due to the unavailability of compatible blood lines for haemodialysis; those weighing more than 25 kg receive haemodialysis.

The standard management of children admitted to LUTH is significantly influenced by the ability of the caregivers to pay for the requested service.

### Definitions

The cause of AKI was taken as the major diagnosis leading to AKI in the child. Sepsis was defined as a systemic inflammatory response due to suspected or proven infection. [13] Systemic inflammatory response was assumed in the presence of white cell count >15×10^3^/mm^3^ at presentation and peripheral temperature >38.0°C persisting for at least 24 hours after presentation. A diagnosis of malaria was confirmed by the presence of asexual forms of plasmodium falciparum on peripheral blood film. Diarrhoea was defined as the passage of three or more loose stools per day. The presence of schistocytosis on blood film, thrombocytopenia and elevated serum creatinine with normal clotting profile was taken as haemolytic uremic syndrome. Nephrotic syndrome was defined as hypoalbuminaemia <25 g/l and spot urine protein-creatinine ratio ≥2 with or without generalized oedema. A diagnosis of acute glomerulonephritis was based on the presence of hematuria with dysmorphic red cells and red cell casts in urine, with or without increased kidney size and echogenicity on ultrasound scan. Hypertension was defined as systolic and or diastolic blood pressure greater than the 95^th^ centile for age, gender and length using normogram published in the fourth report of the National High Blood Pressure Education Group. [14] In all children blood pressure was measured using a mercury sphygmomanometer and an arm cuff with a bladder width that covers at least 40–50 percent of the mid arm circumference [14]; intra-arterial measurement of blood pressure is not available in our hospital.

### Data Management

Analysis of the data was performed using SPSS version 15 (SPSS Inc., Chicago, IL, USA). Continuous data was summarized as mean or median as appropriate, while categorical data was presented as percentages. Prevalence of AKI was calculated as the number of AKI episodes during the study period divided by the total number of children aged one month to 16 years admitted into the paediatrics wards and emergency room of LUTH during the study period. The clinical and laboratory features of children <5 years with AKI were compared with older children using either Student t test or Chi square test as appropriate. Similar comparison was made between children who survived and those who died. A p value of <0.05 was considered to be statistically significant.

## Results

Over 25 months 4 015 children aged between one month and 16 years were admitted into the paediatric wards of LUTH. Of these 69 children were managed for 70 episodes of AKI, giving an AKI prevalence of 17.4 AKI per 1 000 children hospitalized. The median age of the children with AKI was 4.8 years with a range of 0.1–14.4 years. Only two of these children with AKI were admitted into the ICU of the hospital.

### Causes of AKI

The majority (27; 38.6%) of the children had a primary kidney disease, with acute glomerulonephritis and nephrotic syndrome being the most common. Sepsis accounted for AKI in 18 (25.7%) children while severe malaria was the third commonest cause of AKI (8; 11.4%). Gastroenteritis was the underlying disease in 3 children with AKI. With the exception of nephrotic syndrome the majority of children in each category had AKI with maximum category of ‘F’ (Table 1).

**Table 1:** Underlying diagnoses and maximum severity of acute kidney injury in the children studied.

Underlying diseases		Maximum category of AKI		Total, n = 70 (%)
		Risk or Injury	Failure	
Sepsis, n (%)		1 (5.3)	17 (94.4)	18 (25.7)
Primary kidney disease, n (%)		11 (40.7)	16 (59.3)	27 (38.6)
	AGN/RPGN, n (%)	5 (45.5)	6 (54.5)	11.(40.7)
	Nephrotic syndrome, n (%)	5 (62.5)	3 (37.5)	8 (29.6)
	HUS, n (%)	0 (0.0)	4 (100)	4 (14.8)
	Pyelonephritis, n (%)	1 (25.0)	3 (75.0)	4 (14.8)
Cardiac disease^a^, n (%)		1 (25.0)	3 (75.0)	4 (5.7)
Malaria, n (%)		1 (12.5)	7 (87.5)	8 (11.4)
SLE, n (%)		0 (0.0)	3 (100)	3 (4.3)
Others^b^, n (%)		4 (40.0)	6 (60.0)	10 (14.3)

AGN/RPGN: acute glomerulonephritis/rapidly progressing glomerulonephritis; HUS: haemolytic uraemic syndrome; PUV: posterior urethral valves; SLE: systemic lupus erythematosus.

a Cardiac disease: one each of infective endocarditis, complex cyanotic heart disease, severe myocarditis, tricuspid regurgitation post surgery for atrio-ventricular canal defect.

b Others: gastroenteritis (3), HIV/AIDS (2), haemoglobinuria of unknown cause (2), one each of intussusception, dengue haemorrhagic fever and tetanus.

Of the eight children with nephrotic syndrome six had steroid sensitive nephrotic syndrome, and with the exception of one child with AKI RIFLE ‘F’, did not meet any indication for kidney biopsy; these children also had volume-responsive AKI. In the remaining three children with nephrotic syndrome and AKI RIFLE category ‘F’ kidney biopsy could not be done for the following reasons: death during initial hospitalization (1), massive ascites and thrombocytopaenia (1) and financial constraints (1).

In the children with sepsis the median duration of illness before hospitalization was 6.5 (3.1) days. The commonest clinical features in these children before and during hospitalization were fever (19 children), alteration of consciousness (11), convulsion (8), cough and respiratory distress (8), shock (4), bleeding diathesis (2) and hypothermia (2).

### Characteristics of Children with AKI

The features of children with AKI are summarized in Table 2. The male-female ratio was 2.2∶1 with slightly more children younger than 5 years (55.7% versus 44.3%). Fever and a peripheral blood white cell count >15 000/mm^3^ were found in 49 (70.0%) and 35 (50.0%) children respectively at admission into the hospital. In a third of the children diarrhoea was a feature at the time of hospitalization.

**Table 2:** Clinical and laboratory features of children with acute kidney injury.

Characteristics	Alln = 70 (100%)	<5 yearsn = 39 (55.7%)	≥5 yearsn = 31 (44.3%)	p value
Male	48 (68.6)	28 (71.8)	20 (64.5)	0.52
Diarrhoea at presentation	22 (31.4)	18 (46.2)	4 (12.9)	0.00
Fever at presentation	49 (70.0)	30 (76.9)	19 (61.3)	0.16
Oliguria	49 (70.0)	25 (64.1)	24 (77.4)	0.23
Oedema	45 (64.3)	23 (59.0)	22 (71.0)	0.30
Convulsion/loss of consciousness	32 (45.7)	16 (41.0)	16 (51.6)	0.38
Hypertension	35 (50.0)	15 (38.5)	20 (64.5)	0.03
WCC at admission >15 000/mm^3^	35 (50.0)	22 (56.4)	13 (41.9)	0.23
Platelet count <100 000/mm^3^	15 (21.4)	12 (30.8)	3 (9.70)	0.03
Serum potassium ≥5.5mmol/l	29 (41.4)	13 (33.3)	16 (51.6)	0.12
Serum bicarbonate ≤15mmol/l	49 (70.0)	26 (66.7)	23 (74.2)	0.50
AKI at presentation	58 (82.9)	33 (83.3)	25 (79.3)	0.66
Admission eCCl <15ml/min/1.73 m^2^	23 (32.9)	8 (20.5)	15 (48.4)	0.01
Worst eCCl <15ml/min/1.73 m^2^	34 (48.6)	17 (43.6)	17 (54.8)	0.35
RIFLE category ‘F’	49 (70.0)	27 (69.2)	22 (71.0)	0.88
Needed dialysis	22 (31.4)	13 (33.3)	9 (29.0)	0.70
Received dialysis	15 (21.4)	8 (20.5)	7 (22.6)	0.83

WCC: white cell count; eCCl: estimated creatinine clearance; RIFLE: ‘Risk’, ‘Injury’, ‘Failure’, ‘Loss’, ‘End stage’.

The majority (58; 82.9%) of the children had AKI at admission to the hospital, with a third having an estimated creatinine clearance (eCCl) <15 ml/min/1.73 m^2^ at admission. AKI with category ‘F’ was observed in 49 (70.0%) children. Oliguria and severe metabolic acidosis were the commonest features of AKI (70% each). Twenty nine (29; 41%) children had hyperkalemia out of which three children had severe hyperkalemia (serum potassium >7.0 mmol/l).

Both groups of children had similar features except that younger children were more likely to present with diarrhoea and thrombocytopaenia while older children were more likely to have hypertension and an eCCl <15 ml/min/1.73 m^2^at admission.

### Features of Children Needing Dialysis

Twenty two children needed dialysis but only 15 (68.2%) children received dialysis. The indications for dialysis were evidence of symptomatic fluid overload such as pulmonary oedema or intractable hypertension (13), encephalopathy (9), intractable severe metabolic acidosis (5), severe hyperkalemia (3) and bleeding diathesis (2). Most patients had more than one indication. The causes of AKI in the children needing dialysis were sepsis (6), acute glomerulonephritis (4), severe malaria (3) and haemolytic uraemic syndrome (3). Two children had complex cyanotic heart disease while one each had dengue fever, systemic lupus erythematosus, nephrotic syndrome and HIV/AIDS.

### AKI Mortality and Associated Factors

Three children discharged against medical advice leaving 67 episodes of AKI for analysis. Nineteen children (28.4%) with AKI died during hospitalization. Factors significantly associated with mortality were peripheral white cell count >15 000cells/mm^3^ [odds ratio: 5.72 (1.65–19.89)], platelet count <100 000/mm^3^ [odds ratio: 9.56 (2.63–34.77)] and the need for dialysis [odds ratio: 10.04 (2.94–34.33)].

Severity of AKI, presence of hypertension, severe metabolic acidosis, worst eCCl <15 ml/min/1.73 m^2^ and receiving dialysis were not associated with AKI mortality (Table 3).

**Table 3:** Factors associated with acute kidney injury mortality.

Independent variables	Mortality		Odds ratio(95% CI)	P value
	Yes, n = 19 (28.4%)	No, n = 48 (71.6%)		
Age<5 years	13 (68.4)	25 (52.1)	1.99 (0.65–6.11)	0.22
Male	15 (78.9)	32 (66.7)	1.88 (0.53–6.58)	0.32
Hypertension & oedema	7 (36.8)	18 (37.5)	0.97 (0.32–2.92)	0.96
Convulsion/loss of consciousness	10 (52.6)	20 (41.7)	1.56 (0.53–4.53)	0.42
History of reduced urine/Oliguria	16 (84.2)	30 (62.5)	3.20 (0.82–12.52)	0.08
WCC at admission >15 000/mm^3^	15 (78.9)	19 (39.6)	5.72 (1.65–19.89)	0.00
Platelet count <100 000/mm^3^	10 (52.6)	5 (10.4)	9.56 (2.63–34.77)	0.00
Primary kidney disease	6 (31.6)	20 (41.7)	0.65 (0.21–1.99)	0.45
Admission eCCl<15ml/min/1.73m^2^	5 (26.3)	15 (31.3)	0.79 (0.24–2.58)	0.69
Worst eCCl <15ml/min/1.73m^2^	10 (52.6)	21 (43.8)	1.43 (0.49–4.15)	0.51
Serum bicarbonate <15mmol/l	12 (63.2)	34 (70.8)	0.71 (0.23–2.17)	0.54
Serum potassium ≥5.5mmol/l	8 (42.1)	18 (37.5)	1.21 (0.41–3.58)	0.73
RIFLE category ‘F’	14 (73.7)	32 (66.7)	1.40 (0.43–4.58)	0.58
Needed dialysis	12 (63.2)	7 (14.6)	10.04 (2.94–34.33)	0.00
Received dialysis	6 (31.6)	7 (14.6)	2.70 (0.77–9.50)	0.11

WCC: white cell count; eCCl: estimated creatinine clearance; RIFLE: ‘Risk’, ‘Injury’, ‘Failure’, ‘Loss’, ‘End stage’.

## Discussion

The study describes AKI in an unselected group of children admitted to the paediatric wards of a large tertiary hospital in south west Nigeria. The prevalence of AKI defined using paediatric RIFLE (pRIFLE) criteria was 17.4 children per 1 000 children aged one month or older over a 25-month period. The majority had AKI at the time of admission and about a third died.

The present study suggests that the prevalence of AKI in children in Nigeria is high and rising. The AKI prevalence in the present study of 35 cases per year significantly exceeds the 123 cases and 211 cases over 9 and 18 year periods respectively from two large tertiary hospitals in Nigeria. [4], [5] The rising prevalence of AKI has been documented in both developed and developing countries of the world, underlining AKI as a frequent finding in hospitalized children. [1], [3] Although the use of pRIFLE in recent studies and the present one included category ‘R’ and ‘I’, which were not usually recognized as AKI in previous studies, 70% of the children in the present study had the most severe form of AKI, i.e. AKI category ‘F’. This underscores the need for evaluation of kidney function in hospitalized children even in developing countries.

A distinct feature of AKI in the present study is that in the vast majority of children (over 80%) AKI was present at the point of hospitalization and only a few developed AKI while on admission. This observation has been documented in other developing countries such as Turkey, South Africa and Thailand. [1], [8], [15] In sharp contrast, AKI in developed countries develops during hospitalization, the so-called hospital-acquired AKI. [3], [16] Another feature of AKI in our study, which mirrors reports from developing countries, is the high proportion of severe forms of AKI (category ‘F’) in contrast to AKI in developed countries were milder forms are more common. [1], [11] An obvious explanation is the late presentation of sick children to hospital in developing countries. Because healthcare is fee-for-service and the proportion of families with health insurance is small, poverty remains a major hurdle to seeking healthcare early, if any. Another reason for this observation is the detection of AKI only when serum creatinine is elevated or has risen significantly. On the other hand oliguria, a more sensitive marker of AKI, is infrequently used to detect AKI because critically ill children do not have access to the ICU in developing countries; as a result urine monitoring is not commonly performed. The frequent finding of severe category of AKI at admission in the present study argues for community-level interventions in developing countries such as removal of financial barrier to accessing healthcare.

Severe metabolic acidosis was a common feature of children with AKI in the present study, far more prevalent than hyperkalemia and hypertension. In contrast, most studies from developing countries including Nigeria have documented lower frequencies of metabolic acidosis, hyperkalemia and hypertension in children with AKI. [4], [5], [15] The higher frequency of gastroenteritis in these studies may explain these observations. Our finding of high prevalence of severe metabolic acidosis, relative to hyperkalemia and hypertension, may be due to the added burden of sepsis which was the underlying disease in about one third of the children. With a relatively lower incidence of hyperkalemia and hypertension in AKI described in developing countries, it is likely that the need for acute dialysis will be less, creating opportunity for conservative non-dialytic management. [4], [5].

In agreement with previous studies from Nigeria, the disease spectrum causing AKI in children remains the same, i.e. sepsis, primary kidney diseases such as acute glomerulonephritis and nephrotic syndrome and gastroenteritis. [4], [5] However, the present study suggests that the relative contribution of each of these diseases to AKI in children in Nigeria may be changing. For instance, although diarrhoea was a feature at admission in about a third of the children in the present study, the majority of these children had, at admission, features of sepsis, such as fever, convulsion, cough, respiratory distress, leucocytosis and neutrophilia. In addition the frequent observation of hypertension and oedema in the present study, compared to previous studies in which diarrhoea was frequently reported as the cause of AKI, argues against gastroenteritis as the sole underlying cause of the AKI. In these children diarrhoea may be viewed as an additional renal insult in the setting of sepsis or as the initial insult complicated by sepsis. The decline in gastroenteritis-related AKI reflects the world-wide decline in diarrhoea-related deaths which has been partly attributed to better sanitation and increased home use of oral rehydration solution. Recent studies from developing countries such as Turkey and Thailand support this observation. [1], [15] Similarly, the relative contribution of malaria to AKI may be declining; the present study indicates a significantly lower frequency of malaria among children with AKI in Nigeria compared to previous reports. [4], [5] The smaller contribution of malaria in the present study may reflect the reported decline in malaria-related deaths and the increasing contribution of multi-systemic disease and co-morbidities, such as sepsis, systemic lupus erythematosus, HIV/AIDS and cardiac diseases to AKI. [17] Inexplicably but consistent with studies from Nigeria haemolytic uremic syndrome was infrequently reported as a cause of AKI. [4], [5] Under-diagnosis may be a reason for this.

In the present study, it may be argued that sepsis is the commonest cause of AKI. Although it ranked second to primary kidney disease, a significant number of the children with other underlying causes had features suggestive of sepsis. Indeed fever with a peripheral temperature more than 38°C and leucocytosis greater than 15 000 cells/mm^3^ were present at admission to the hospital in two thirds and one half of the children respectively. In children with sepsis, AKI may arise from renal hypoperfusion, tubular necrosis and drug nephrotoxicity.

AKI still carries high mortality underscoring its recent recognition as a marker of illness severity among children and adult admitted to the hospital. [11], [18] Our AKI-mortality of about 28% is similar to the findings of several studies from different parts of the world and different hospital settings. [3], [7] Compared to previous studies from sub Saharan Africa the AKI mortality in the present study is significantly lower than the reported 37–46% mortality. [4], [5], [6] An obvious explanation is the increased access to renal replacement therapy in the present study which, at 68%, was significantly higher than the rates documented in previous studies from most of sub Saharan Africa. [4], [5], [6] Although more children in previous studies needed dialysis than the present study, inclusion of serum creatinine and urea values as indications for dialysis in these studies may have inflated these numbers. However, as evident in our study mortality from AKI may not be solely due to AKI. Indeed features of severe AKI such as oedema, hypertension, hyperkalemia, metabolic acidosis or eCCl <15 ml/min/1.73 m^2^ were not associated with increased odds of AKI mortality in the present study. Specifically, the presence of both hypertension and oedema, a surrogate of fluid overload was not predictive of mortality. Rather, markers suggestive of sepsis such as leucocytosis and thrombocytopaenia were associated with significant odds of AKI mortality. This buttresses the observation by other workers that mortality in AKI may also imply failure of multiple organs and the need for management in intensive care units. [7], [8], [19] Consistent with published literature children with single disease entities like primary kidney disease were likely to survive but the difference was not significant in the present study. [20], [21].

Our study has some limitations. Although, the classification of the cause of AKI as the major underlying disease in the child did not specify the pathogenetic mechanism leading to AKI, it recognizes that more than one pathophysiologic process may lead to AKI in a particular child. For instance, in children with posterior urethral valves, the presence of febrile urinary tract infection and the use of antibiotics may represent additional injury to the kidneys. The classification employed in the present study also flags children at high risk of AKI, allowing more frequent evaluation for AKI. The preponderance of severe forms of AKI suggests that less severe cases may have been missed. This is likely because at admission most children with AKI were detected because of elevated serum creatinine rather than due to oliguria. This is not unexpected when severely ill children are managed outside the ICU; accurate urine monitoring is infrequently achieved in the general ward due to the increase patient to nurse ratio. Nonetheless, it suggests that the high prevalence of AKI in the present study may be an underestimation of the true prevalence of AKI in hospitalized children. Most cases of sepsis in the study were not culture proven, however the preponderance of clinical features suggestive of multi-system involvement in the disease process, marked leucocytosis and fever suggest a sepsis syndrome.

In conclusion the present study observes that the prevalence of AKI among hospitalized children in Nigeria is high and rising. Primary kidney disease and sepsis are the commonest cause of AKI; the contribution of gastroenteritis and malaria is declining. AKI-related mortality remains high with requirement for dialysis, leucocytosis and thrombocytopaenia as associated factors.
